# Human Organoids as a Promising Platform for Fighting COVID-19

**DOI:** 10.7150/ijbs.64993

**Published:** 2022-01-01

**Authors:** Dong Chen, Xi Su, Haibo Chen, Siyan Chen, Yongsheng Zhao, Wei Wei

**Affiliations:** 1 Department of Thyroid and Breast Surgery, Peking University Shenzhen Hospital, Shenzhen 518036, China.; 2 Department of Nuclear Medicine, Peking University Shenzhen Hospital, Shenzhen 518036, China.; 3 College of Life Sciences and Oceanography, Shenzhen University, Shenzhen 518060, China.

**Keywords:** organoid, SARS-CoV-2, COVID-19, infection, drug screening

## Abstract

The coronavirus disease 2019 (COVID-19) global pandemic evoked by the severe acute respiratory syndrome coronavirus 2 (SARS-CoV-2) has triggered a major public health problem with significant morbidity and mortality. Understanding the pathogenesis and molecular mechanisms underlying this novel virus is crucial for both fundamental research and clinical trials in order to devise effective therapies and vaccination regimens. Basic research on SARS-CoV-2 largely depends on *ex vivo* models that allow viral invasion and replication. Organoid models are now emerging as a valuable tool to investigate viral biology and disease progression, serving as an efficient platform to investigate potential therapies for COVID-19. Here, we summarize various human stem cell-derived organoid types employed in SARS-CoV-2 studies. We highlight key findings from these models, including cell tropisms and molecular mechanisms in viral infection. We also describe their use in identifying potential therapeutic agents against SARS-CoV-2. As more and more advanced organoids emerge, they will facilitate the understanding of disease pathogenesis for drug development in this dreaded pandemic.

## Introduction

The coronavirus disease 2019 (COVID-19) pneumonia epidemic caused by the severe acute respiratory syndrome coronavirus 2 (SARS-CoV-2) has become the worst public health threat in the current century. Treatment strategies for prevention and intervention are a matter of urgency. Although many clinical trials are currently underway, preclinical research on *in vitro* models is also needed to allow us to better understand virus infections and to test drugs and vaccines for safety and efficacy.

Until now, there has only been a limited number of animal models available in SARS-CoV-2 research. The most susceptible animals to this virus are cats 1, ferrets 1, 2, rhesus macaques 3, cynomolgus macaques 4, and golden hamsters 5. SARS-CoV-2 utilizes angiotensin converting enzyme 2 (ACE2) for cellular entry and the cell surface transmembrane serine protease 2 (TMPRSS2) for spike protein priming 6, 7. Transgenic mice expressing human ACE2 can be a good option to study COVID-19 8. Nevertheless, animal-based studies are generally expensive and lengthy, and fail to fully mimic human physiology due to species specificity.

Classic cell lines are a great alternative to animal models. In fact, much of the insight on the pathogenesis and drug responses of SARS-CoV-2 has been obtained using numerous cell lines, such as Vero (kidney epithelial cells), Caco2 (intestinal cells), Calu3 (pulmonary cells), and Huh7 (hepatic cells) 7, 9, 10. However, cell lines are mostly of malignant origin and cannot simulate cell-cell and cell-matrix interactions. The *in vivo* infection status and pathological features may also be poorly recapitulated in these two-dimensional (2D) cultures.

A range of recent studies on COVID-19 highlights the value of more physiological *in vitro* models, called organoids. Organoids are miniaturized, three-dimensional (3D) tissue models that derived from induced pluripotent stem cells (iPSCs), embryonic stem cells (ESCs), or multipotent adult stem cells (ASCs) 11, 12. Importantly, they consist of various cell types and faithfully recapitulate the essential structure and physiological characteristics of the parental organ in a dish 13, 14. Organoids are powerful tools for disease modeling *in vitro*, providing a more flexible, efficient, and large-scale drug screening platform than *in vivo* models. They are also ethically humane tools used to study physiology and disease, including viral infections.

## Organoids in virology

In virology, organoids are particularly useful as they permit studies on viruses that are difficult to cultivate. Organoid models have been shown to be an excellent model to study viral infections and host-virus interactions 15-23. Generating various organoid models from human tissues has become more important in studying viral infections as patients show signs of systemic symptoms in addition to respiratory infection.

Most of airborne viruses enter human body by infecting epithelial cells. Organoids derived from oral mucosa, airway, lung, and intestines have been used to model viral infection and replication. Organoids derived from human oral mucosal maintain morphologic and functional characteristics of their parental tissues, and have been shown to be productively infected by human papillomavirus (HPV) and herpes simplex virus (HSV) 16. Human airway organoids have emerged as a valuable tool to assess the susceptibility of animal influenza viruses 23, 24. Zhou, *et al.,* proved that the differentiated human airway organoids are susceptible to the human-​infective influenza viruses H7N9 and H1N1, while avian H7N2 and swine H1N1 influenza viruses showed lower replication levels 23. Another study used human airway organoid cultures to measure replication competence, tissue tropism and host response to human influenza virus 24. Moreover, Sachs, *et al.,* used human airway organoids to show dramatic epithelial remodeling after respiratory syncytial virus (RSV) infection 21. Noroviruses are a major cause of gastroenteritis in the world. Human stem cell-derived intestinal organoids have become the first *ex vivo* infection model to support noroviruses replication 18. Indeed, the human intestinal organoid cultivation system recapitulates the physiologically active intestinal epithelium, and allows studies of norovirus replication efficiency *in vitro* 17. This model was also used to confirm that the intestines are a target organ for Middle East respiratory syndrome coronavirus (MERS-CoV) 25.

During the 2015 Zika virus (ZIKV) outbreak, human iPSC-derived cerebral organoids were adopted to provide proof that this virus selectively replicates in the developing brain, preferentially infecting neural cell precursors, leading to congenital abnormalities including microcephaly 22, 26, 27. These studies provide the explanation as to why the virus has a greater detrimental effect on the fetal brain compared to the postnatal brain. By using cortical organoids to infect cytomegalovirus, Sison, *et al.,* found organoid structure alterations and disruptions in specific marker expression 28. Furthermore, human liver organoids also emerged as a promising personalized infection model in the treatment of hepatitis B virus (HBV). By co-culture of human iPSC-derived endodermal, mesenchymal and endothelial cells with a chemically defined medium in a 3D system, Nie and colleagues generated the functional liver organoids that can inherit the genetic background of donors and reproduce host-virus interactions by simulating HBV propagation and the virus-induced hepatic dysfunction 29.

## Human organoids in studying SARS-CoV-2 infection

**Figure 1:**
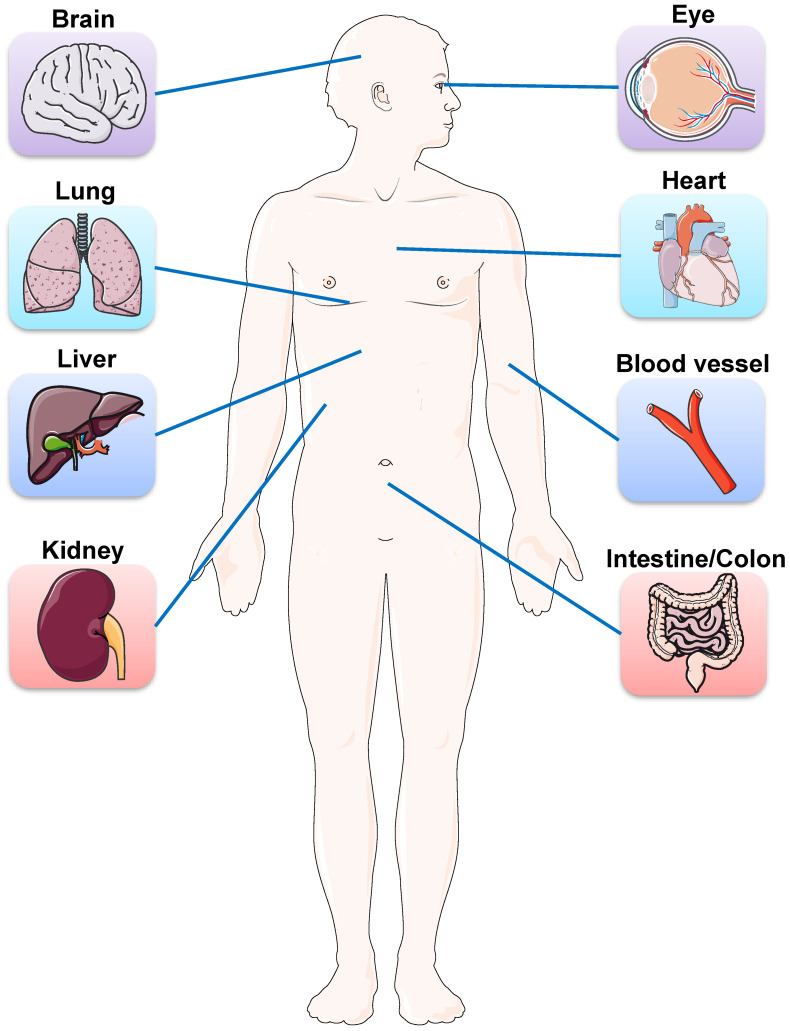
Schematic representation of the main organs affected by SARS-COV-2. Besides lung injury caused by SARS-CoV-2, symptoms have also been noted in multiple other organs, including brain, eye, heart, liver, intestine, kidney, and blood vessels.

**Figure 2:**
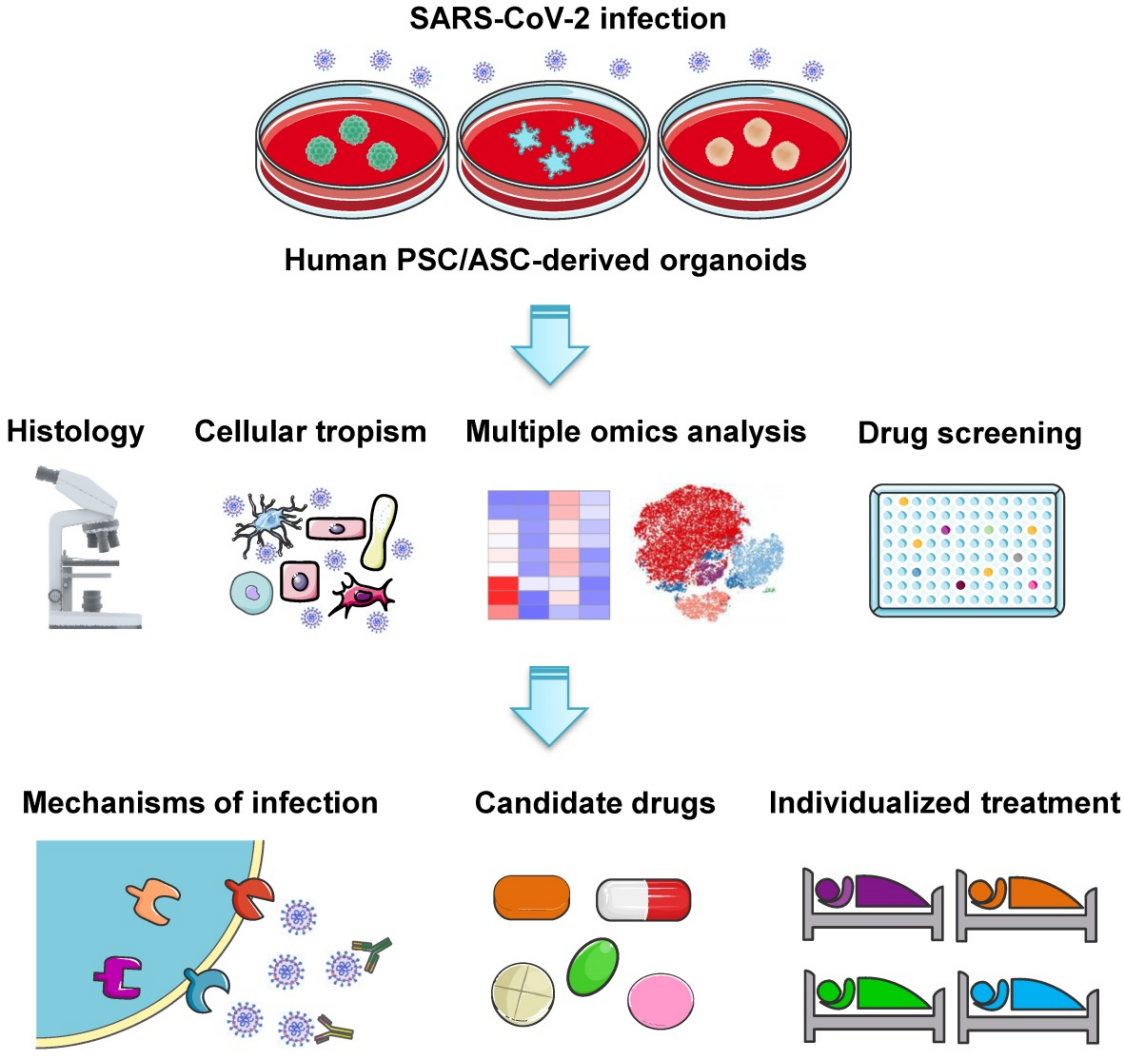
Common analyses and key applications of human PSC- and ASC-derived organoid platforms in COVID-19 research. Various organoids derived from PSCs or ASCs have been established to study SARS-CoV-2 infection. Organoid models are commonly used to investigate SARS-CoV-2 tropism and COVID-19 pathophysiology across different organs, as well as to verify the safety and efficacy of candidate drugs and screen new therapeutic strategies. Furthermore, patient-derived organoids may potentially serve as a platform to test the efficacy of antiviral drugs for individual patients.

Besides lung injury caused by SARS-​CoV-2, symptoms have also been noted in multiple other organs, including brain, liver, intestine, kidney, eye, heart, and blood vessels (Figure 1). Many research groups have utilized organoid technologies to understand the tissue tropism and cellular response of SARS-​CoV-2 and the damage caused. Organoids can be established from either human pluripotent stem cells (PSCs) including ESCs and iPSCs, or multipotent adult tissue stem cells (ASCs). SARS-CoV-2 infection and replication have been studied in PSC- and ASC-based organoids derived from a wide range of organs (Table 1). Organoid models are proving their worth in verifying the safety and efficacy of antiviral therapies as these *in vitro* models can exploit a priori knowledge on virus biology, and allow for the testing of well-known viral inhibitors and the discovery of new drugs (Figure 2).

### Lung organoids

SARS-​CoV-2 has been demonstrated to employ two key host proteins, ACE2 and TMPRSS2, to bind and infect host cells 6, 7. ACE2 and TMPRSS2 have been shown to be highly expressed in human airway organoids 23, 30, indicating that they are suitable for SARS-​CoV-2 research. Air-liquid interface (ALI) cultures of human airway organoids were readily infected by the addition of SARS-CoV-2 to the apical side 31. The virus infection was observed in ciliated cells but not in goblet cells, suggesting that ciliated cells are the main targets of the virus 31. In a recent study, alveolar cells, basal cells, and rare neuroendocrine cells were grown from human fetal lung bud tip progenitor organoids 32. Most of the infected cells were alveolar type II cells, and a low dose of interferon lambda 1 (IFN-λ1) was shown to reduce viral replication. Therefore, ciliated cells and alveolar cells are susceptible to SARS-CoV-2 infection, and ciliated cells could be infected prior to the alveoli 32. Ebisudani *et al.,* established an efficient ASC-based alveolosphere culture system. These alveolospheres express ACE2 at both RNA and protein levels, and maintain the robust infection efficiency of SARS-CoV-2 even after long-term cultivation 33. Salahudeen, *et al.,* generated human distal lung organoids with apical-out morphology to present ACE2 on the exposed external surface, allowing them to be infected by SARS-CoV-2 34. Contrary to Lamers's work 31, goblet cells, but not ciliated cells, were infectable in their organoid cultures 34. Additionally, ASC-derived human lung organoids consisting of both proximal and distal airway epithelia have been established, and the proximal airway epithelium has been shown to be more permissive to SARS-CoV-2 infection 35. Han, *et al.,* demonstrated SARS-CoV-2 entry and infection in human ESC-derived lung organoids, which are mainly composed of alveolar type-I (AT1) cells, alveolar type-II (AT2) cells, stroma cells, neuroendocrine cells, and airway epithelial cells 36. Gene ontology analysis of infected lung organoids revealed that most upregulated genes were associated with immune response. Several chemokines and cytokines, including tumor necrosis factor (TNF), interleukin (IL), as well as nuclear factor kappa beta (NF-κB) were observably upregulation 36, 37. A progressive increase of cell death in lung organoids became evident at 72 hours post infection, and this was further validated by immunostaining experiments 37.

Lung organoids derived from human pluripotent stem cells (hPSCs) were permissive to mass production, cryopreservation, and genetic manipulation 38. They also allowed researchers to assess the antiviral effects of COVID-19 candidate drugs 33, 36, 37, 39, 40. Remdesivir, a nucleotide analogue prodrug used to inhibit viral replication, has been shown to greatly reduce the production of infectious virus particles in both human airway organoids and alveolar organoids, while camostat (a TMPRSS2 inhibitor) has been shown to slightly decrease the production of the virus in airway organoids but not alveolar organoids 37. Remdesivir has also been shown to inhibit SARS-CoV-2 replication in human alveolospheres at the concentration comparable with the circulating drug level 33. Suzuki, *et al.,* found a reduction of viral copy number in the infected bronchial organoids with treatment of camostat 40. Neutralizing antibody CB6, one of the promising neutralizing antibodies to treat COVID-19 41, significantly decreased the production of infectious viral in lung organoids 37. In addition, pre-treatment of lung organoids with imatinib, chloroquine, mycophenolic acid (MPA), and quinacrine dihydrochloride (QNHC) effectively blocked the ACE2 cleavage site, suggesting a potential role in decreasing SARS-CoV-2 infection 36. Human lung organoids have also been used to test the efficacy of candidate antiandrogenic drugs, such as finasteride, ketoconazole, and dutasteride, in resisting SARS-CoV-2 infection. These drugs have been certified to downregulate the expression of ACE2 in lung organoids, and therefore to reduce susceptibility to the virus 39. Overall, human lung organoids can serve as an excellent model to investigate SARS-CoV-2 infection and to screen and discover candidate COVID-19 therapeutics.

### Intestinal organoids

Apart from respiratory illnesses, gastrointestinal symptoms have also been identified in a subset of patients 42, indicating that the gastrointestinal tract may be a potential entry route for SARS-CoV-2. Many kinds of bats are disreputable carriers of zoonotic viruses that occasionally spread to humans, including coronaviruses. Based on the close relation of SARS-CoV-2 to SARS-CoV identified in bat species, intestinal organoids were established from human and the horseshoe bat* Rhinolophus sinicus*, and their sensitivities to SARS-CoV-2 infection were examined for the first time 43. Both human and bat intestinal organoids were successfully infected by SARS-CoV-2 isolated from COVID-19 patients, leading to progressive cytopathic effects after virus replication. In addition, the expression of ACE2 and TMPRSS2 were significantly increased upon induction of organoid differentiation, indicating that SARS-CoV-2 infection occurs in differentiated cells 43. These findings are consistent with an independent study that revealed infection and replication of the virus in the enterocytes of human small intestinal organoids 31. As expected, ACE2 is highly expressed in differentiated enterocytes. Infected enterocytes produced viral particles, and elicited a significant upregulation of viral response genes, probably via cytoplasmic sensing of the viral RNA genome 31. Human ESC-derived colonic organoids were also readily infected by SARS-CoV-2 36. Transcriptional profiling of colonic organoids indicated that enterocytes were the cell types most susceptible to SARS-CoV-2-entry virus 36. In addition, human duodenum- and ileum-derived organoids were permissive to SARS-CoV-2 infection, and the two mucosa-specific serine proteases, TMPRSS2 and TMPRSS4, activated SARS-CoV-2 spike protein and facilitated virus entry into host cells 44. These studies used human intestinal organoids to support clinical evidence that the gastrointestinal tract is a possible transmission route of SARS-CoV-2.

Several studies have employed intestinal organoids to test drug candidates that might ameliorate gastrointestinal illnesses in the clinic. Krüger and colleagues 45 utilized ESC-derived intestinal organoids to test the efficiency of three candidate drugs: remdesivir 46, famotidine 47 and EK1 48. Results of this study indicate that remdesivir and EK1, but not famotidine, inhibited viral infection and replication 45. Similar to the drug reaction in lung organoids, the three drug candidates imatinib, mycophenolic acid and quinacrine dihydrochloride also block SARS-CoV-2 infection in colonic organoids 36. Furthermore, using human colon-derived organoids, Stanifer, *et al.,* demonstrated that pre-treatment of colon-derived organoids with both type I IFN (IFN-β1) and type III IFN (IFN-λ) significantly blocked SARS-CoV-2 infection and this was related to a reduction in viral genome copy number 49.

### Brain organoids

A mounting number of COVID-19 cases exhibit neurologic symptoms and neuropsychiatric disorders 50, 51, suggesting that the central nervous system (CNS) may be vulnerable to this virus. Human iPSCs-derived brain organoids have allowed several groups to independently examine the neurotropism and neurotoxic effects of SARS-CoV-2 in this pandemic. Ramani, *et al.,* revealed that SARS-CoV-2 could infect human brain organoids within two days of virus exposure 52. Interestingly, no productive replication of the virus could be observed in neural cells, supporting the hypothesis that SARS-CoV-2 can use the CNS as a long-term reservoir 53. However, neurodegeneration-like effects consisting of extensive cell death and hyperphosphorylation, were observed in these neurons infected by SARS-CoV-2, associated with misallocation of the structural protein Tau 52. Similarly, several studies also found that neurons including neural progenitor cells (NPCs) and mature cortical neurons in these organoids were observed to be infected by SARS-CoV-2 54, 55. On the contrary, Bullen,* et al.,* found an increased accumulation of viral particles in neuronal cells of brain organoids infected with SARS-CoV-2, indicating an active infection and replication of the virus in neurons 56. These contradictory results may be due to differences in experimental conditions between studies by different teams, such as the time of SARS-CoV-2 infection and the adoption of iPSC-derived organoids at different differentiation and developmental stage.

A recent study systematically tested SARS-CoV-2 infection in various region-specific brain organoids of the hypothalamus, midbrain, hippocampus, and cortex 57. Intriguingly, the choroid plexus epithelium expressed in hippocampal organoids were more vulnerable to the virus than neurons, suggesting that the choroid plexus epithelium is probably the gateway for the entry of the SARS-CoV-2 into the human brain. This possibility is supported by recent findings that show abundant expression of ACE2 and TMPRSS2 in choroid plexus epithelial cells 58, 59. The productive SARS-CoV-2 infection of choroid plexus organoids led to cell death, transcriptional upregulation of inflammatory genes, and functional deficits 57. Pellegrini, *et al.,* also found a stronger tropism of SARS-CoV-2 towards the choroid plexus than neurons within choroid plexus organoid cultures 59. The infection by SARS-CoV-2 damages choroid plexus epithelial cells, leading to leakage of the blood-cerebrospinal fluid barrier which plays a vital role in preventing the entry of pathogens, immune cells, and cytokines into the cerebrospinal fluid and brain 59.

Human PSC-based brain organoids have become permissive to high-throughput drug screenings, consequently accelerating the discovery or repurposing of drugs for preventing and treating CNS related COVID-19 symptoms. Sofosbuvir, an FDA (US Food and Drug Administration)-approved nucleotide polymerase inhibitor 60, was used as a treatment for SARS-CoV-2 infection. Notably, this drug has been shown to decrease viral accumulation and reduce neuronal death in brain organoids, highlighting the potential abirritation for neurological symptoms 61. Song, *et al.,* found metabolic changes in SARS-CoV-2-infected neurons in brain organoids, but no evidence of type I interferons (IFN-I) response was detected 54. IgG antibodies against SARS-CoV-2 present in the cerebrospinal fluid from a patient hospitalized with COVID-19 were able to prevent virus infection of brain organoids 54. Overall, these studies exhibit great potential of human PSC-based brain organoids for probing the infection of SARS-CoV-2 virus in the CNS.

### Liver organoids

As liver damage has been observed in patients with COVID-19 62, experimental platforms including human liver ductal organoids, hepatocyte and cholangiocyte organoids have been used as models of SARS-CoV-2 infection. Yang, *et al.,* established adult liver hepatocyte and cholangiocyte organoids from bile duct epithelial cells and found a high expression of both ACE2 and TMPRSS2 in these organoid derivatives 63. Liver hepatocyte and cholangiocyte organoids are permissive to both SARS-CoV-2 pseudo-entry virus and SARS-CoV-2 virus infection. SARS-CoV-2 infection caused significant expression of chemokines and upregulation of inflammatory pathways, as also seen in autopsy samples from patients with COVID-19 63. Zhao, *et al.,* reported a liver ductal organoid model generated from liver bile duct-derived progenitor cells grown in a 3D culture system 64. Cholangiocytes in human liver ductal organoids express ACE2 and TMPRSS2 that enable SARS-CoV-2 infection. Infected cells in liver ductal organoids overexpressed chemokines, formed syncytia and underwent extensive apoptosis with injury of the bile acid transporting functions, resulting in the accumulation or leakage of bile acid and a series of clinical symptoms in COVID-19 patients 64. These observations suggest that the hepatic injury caused directly by SARS-CoV-2 infection should also be taken into account in treating COVID-19 patients. Together, hPSC-derived liver organoids provide a valuable platform for understanding the tropism and pathogenesis of SARS-CoV-2 and discovering prospective anti-viral therapeutics.

### Kidney organoids

Given the presence of renal manifestations such as proteinuria, hematuria, and other classic symptoms in severe COVID-19 patients 65, 66 and the detection of this virus in the urine of infected individuals 67, human kidney organoids have also been tested as a model of SARS-CoV-2 infection. Kidney organoids were recently established from human ESCs, and can be directly infected by SARS-CoV-2 68. Consistent with widely expressed ACE2 in human kidney biopsies, ACE2 is expressed in podocytes and proximal tubular cells of human kidney organoids 68. Another study generated long term cultures of organoids from human kidney proximal tubular epithelial cells and found that ACE2 was more highly expressed in 3D culture than in 2D cultured cells 69. To confirm whether SARS-CoV-2 invades renal cells via ACE2, human recombinant soluble ACE2 (hrsACE2) was added to competitively bind to the virus rather than host cells 68, 70. As a result, hrsACE2 significantly decreased SARS-CoV-2 infection of kidney organoids in a dose-dependent manner without causing cell toxicity. Wysocki, *et al.,* also proved the neutralizing effect of their modified long-acting ACE2 variants to resist SARS-CoV-2 in human kidney organoids 71. Interestingly, hrsACE2 could markedly improve the effect of remdesivir in SARS-CoV-2 infection in kidney organoid cultures 70. These observed efficacies of ACE2 in kidney organoids encourage further clinical investigation of this drug alone or in combination with other drugs.

### Cardiac and vascular organoids

Cardiovascular complications are another common condition in patients with severe COVID-19 and increase the risk of mortality 72-74. Human PSC-based cardiac organoids 75, 76 and vascular organoids 68 have been established as potential *in vitro* models to study SARS-CoV-2 infection in the cardiovascular system. The high expression of ACE2 has been observed in cardiomyocytes, endothelial cells, vascular smooth muscle cells, and pericytes lining blood vessels 77, 78.

Mills *et al*., developed a high-throughput hPSC-cardiac organoid platform, using it to screen 105 small molecules with regenerative potential. These organoids could also be a feasible platform to study virus infection and screen antiviral drugs 79. Recently, by using human cardiac organoids, Mills, *et al*., showed that bromodomain and extraterminal family inhibitors (BETi) reduced ACE2 expression, decreased transcription of genes in the viral response, and blocked SARS-CoV-2 infection of cardiomyocytes and inflammation-induced cardiac dysfunction 76. By using iPSC-derived blood vessel organoids, Monteil, *et al*., validated that SARS-CoV-2 was able to infect human blood vessels 68. Importantly, the virus replication was detected in these organoids after infection. The addition of clinical-grade hrsACE2 dramatically reduced SARS-CoV-2 infections of human blood vessel organoids 68. Consistently, organoids infected with mixtures of SARS-CoV-2 virus particles and variable concentrations of hrsACE2 have markedly decreased levels of intracellular viral RNA 68. These findings may explain how the virus spreads through the body and leads to organ damage in severely ill individuals.

### Eye organoids

Recent clinical evidence has shown that a minority of COVID-19 patients present with ocular symptoms 80, 81. Importantly, SARS-CoV-2 entry factors ACE2 and TMPRSS2 have been found to be expressed in the limbal, corneal, and conjunctival epithelium of the eye and their organoid derivatives 82-85. Also, SARS-CoV-2 viruses have been detected in the conjunctiva of infected rhesus monkeys 86. These findings indicate that ocular surfaces may serve as an additional entry vector for SARS-CoV-2. Therefore, eyes are at risk for infection by SARS-CoV-2 and are a route warranting protection. Human iPSC-derived whole-eye organoid models, which are comprised of cells from the retina, cornea, ciliary margin, retinal pigment epithelium, lens and iris as well as cell monolayers grown from limbal, corneal and conjunctival epithelium, have been established to study SARS-CoV-2 ocular infection 84. In fact, the corneal limbus seems to be the most susceptible to infection, consistent with its high expression of ACE2 and TMPRSS2, essential proteins that mediate SARS-CoV-2 viral entry. This means that the cornea may be the entrance and proliferation site of virus infection. Although IFN-I and IFN-III were not detected, human eye organoids mounted a significant inflammatory response indicative of NF-κB signaling 84. Similar findings were obtained from ocular biopsies from human beings infected with SARS-CoV-2, supporting the utility of eye organoids to study SARS-CoV-2 infection and identify prophylactics that may protect the eyes from infection 84. Future studies are required to better clarify how infection in the eye may lead to transmission to other regions of the body.

## Limitations and perspectives

Exploring the life cycle of SARS-CoV-2 and unearthing the efficacy of drugs can be made more objective by using organoid models. Organoids models can simulate the pathology of COVID-19 in corresponding tissues, and provide a valuable platform for unraveling virus infectivity, tropisms, replication kinetics and potential treatments 87, 88. Although a range of new studies demonstrated many more advantages of human organoids, these models still have some limitations. The microenvironment of organoids differs from the native organ to some extent due to the absence of additional cell types such as fibroblasts, vascular cells, neural cells and immune cells. In general, iPSC-derived organoids can contain mesenchymal lineages such as fibroblasts, while ASC-derived organoids consist exclusively of epithelial cells 89. Moreover, organoid models cannot fully represent the systemic symptoms associated with whole body responses to this disease. This means virology research using organoids will still need to be validated in animal models and clinical studies. Anyhow, it is worth noting that organoids exhibit greater complexity and resemble the true organ more closely than any other 2D cultured cells.

Immune cells play a vital role in the fight against SARS-CoV-2 infection 90. Organoids derived from non-small-cell lung cancer and colorectal cancer have been co-cultured with T cells from individual patients 91, 92. The co-culture system showed successful antigen presentation of tumor organoid cells to T cells, which subsequently became activated 92. Immune co-cultured organoids can also be applied to virus infection research to understanding COVID-19 immune responses. Furthermore, several studies have reported promising organoid-based findings, including the bioengineering of a scaffold-guided functional intestine using a bioreactor 93, 94 and organoids-on-a-chip 95. Such physiologically relevant culture systems, as well as functional simulation of immune responses in bioengineered organoids, will open up new perspectives for disease modelling to help fight the current pandemic.

## Figures and Tables

**Table 1:** List of human PSC-derived organoids used to study SARS-CoV-2 infection

Organoid type	Origin	Key points	Drug candidates
Lung organoids/Airway organoids/Alveolar organoids	Human embryonic stem cells	SARS-CoV-2 entry and infection in lung organoids, which are mainly composed of alveolar type-I, alveolar type-II, stroma, neuroendocrine, and airway epithelial cells 36; SARS-CoV-2 were shown to infect ciliated, club, and alveolar type 2 cells in airway and alveolar organoids, and induce the downregulation of the metabolic processes and the upregulation of immune response 37; Remdesivir was shown to greatly reduce the production of infectious virus particles in airway and alveolar organoids 37; Androgen signaling inhibition reduces SARS-CoV-2 infection in lung organoids 39.	Remdesivir; Imatinib; Camostat; Mycophenolic acid (MPA); Quinacrine dihydrochloride (QNHC); Chloroquine; Neutralizing antibodies; Dutasteride; Ketoconazole; Finasteride.
Intestinal organoids/Colonic organoids	Human embryonic stem cells	Colonic organoids are permissive to infection by SARS-CoV-2 36; Enterocytes express ACE2 and are the cell types most susceptible to SARS-CoV-2 infection 36; Remdesivir and EK1 inhibited SARS-CoV-2 infection and replication in intestinal organoids 45.	Remdesivir; Imatinib; Mycophenolic acid (MPA); Quinacrine dihydrochloride (QNHC); Famotidine; EK1.
Brain organoids/Choroid plexus organoids	Human embryonic stem cells; Human induced pluripotent stem cells	Brain organoids are permissive to infection but do not support active viral replication 52; Neuronal infection was inhibited by blocking ACE2 with antibodies or by administering cerebrospinal fluid from a COVID-19 patient 54; SARS-CoV-2 can directly target cortical neurons and neural progenitor cells in brain organoids 55; Extensive viral protein expression and infectious viral particles were detected in brain organoids infected with SARS-CoV-2 56; Choroid plexus organoids are permissive to productive infection, leading to transcriptional upregulation of inflammatory genes 57; SARS-CoV-2 infects choroid plexus, leading to damage of this brain barrier 59; Sofosbuvir protects brain organoid from SARS-CoV-2 infection 61.	IgG antibodies present in the cerebrospinal fluid of COVID-19 patients; Sofosbuvir.
Liver organoids	Human induced pluripotent stem cells	Human hepatocyte and cholangiocyte organoids are highly permissive to SARS-CoV-2 infection 63; Liver organoids show similar chemokine response as COVID-19 patients 63.	/
Kidney organoids	Human embryonic stem cells	Kidney organoids express ACE2 and TMPRSS2 68; SARS-CoV-2 can directly infect kidney organoids 68; Human recombinant soluble ACE2 reduce SARS-CoV-2 infection in kidney organoids 68, 71; Combination therapy using Remdesivir with recombinant soluble ACE2 reduces virus entry and replication 70.	Human recombinant soluble ACE2; Remdesivir.
Cardiac organoids	Human embryonic stem cells	BET inhibitors reduce ACE2 expression, decrease transcription of genes in the viral response, and block SARS-CoV-2 infection of cardiomyocytes and inflammation-induced cardiac dysfunction 76.	Bromodomain and extraterminal family inhibitors (BETi).
Blood vessel organoids	Human induced pluripotent stem cells composed of vascular networks of endothelial cells	Human blood vessel organoids were highly susceptible to SARS-CoV-2 infection can be inhibited in an ACE2-dependent manner 68	Human recombinant soluble ACE2.
Eye organoids	Human induced pluripotent stem cells Human embryonic stem cells	The limbus was most susceptible to SARS-CoV-2 infection due to the high expression of ACE2 and TMPRSS2 84; Interferon response type I and III is suppressed upon infection with SARS-CoV-2 84.	/

**Table 2:** List of ASC-derived organoids used to study SARS-CoV-2 infection

Organoid type	Origin	Key points	Drug candidates
Airway organoids/Bronchial organoids/Distal lung organoids/Fetal lung bud tip organoids/Alveolospheres	Adult small airway donor; Normal human bronchial epithelial cells; Distal airway cells from patient lung tissue; Fetal lung donors; Normal lung tissues of patients	Ciliated cells and alveolar cells are susceptible to SARS-CoV-2 infection, and ciliated cells could be infected prior to the alveoli 31, 32; Human alveolospheres express ACE2 and allow robust SARS-CoV-2 infection 33; Remdesivir inhibits SARS-CoV-2 replication in alveolospheres 33; Distal lung organoids with apical-out morphology to present ACE2 on the exposed external surface, allowing SARS-CoV-2 infection 34; Goblet cells but not ciliated cells were infectable in organoid cultures 34; ASC-derived lung organoids consisting of both proximal and distal airway epithelia, and the proximal airway epithelium are more permissive to SARS-CoV-2 infection 35; ACE2 and TMPRSS2 are highly expressed in bronchial organoids 40; A reduction of viral copy number in the infected bronchial organoids with treatment of camostat 40; Not only intracellular viral genome, but also progeny virus, cytotoxicity, pyknotic cells, and moderate increases of the type I interferon signal can be observed after SARS-CoV-2 infection 40.	Remdesivir; Lopinavir; Nelfinavir; Camostat
Intestinal organoids/Colonic organoids	Normal intestinal and colonic samples from patients	Enterocytes produced infectious viral particles, and induced a generic viral response 31; SARS-CoV-2 was able to infect human intestinal organoids, leading to progressive cytopathic effects with virus replication over time 43; Duodenum- and ileum-derived organoids are permissive to infection and support robust viral replication 44; Pre-treatment of colon-derived organoids with both IFN-β1 and IFN-λ significantly blocked SARS-CoV-2 infection and this was associated with a decrease in viral genome copy number 49.	/
Liver ductal organoids	Primary bile ducts isolated from human liver biopsies	ACE2 and TMPRSS2 are involved in viral entry 64; SARS-CoV-2 infection impairs bile acid transportation functions 64.	/

## Abbreviations

2D: two-dimensional
3D: three-dimensional
ACE2: angiotensin-converting enzyme 2
ALI: air-liquid interface
ASC: adult stem cell
AT1: alveolar type-I
AT2: alveolar type-II
BETi: bromodomain and extraterminal family inhibitors
CNS: central nervous system
COVID-19: coronavirus disease 2019
ESC: embryonic stem cell
FDA: Food and Drug Administration
hrsACE2: human recombinant soluble angiotensin-converting enzyme 2
HBV: hepatitis B virus
hPSC: human pluripotent stem cell
HPV: human papillomavirus
HSV: herpes simplex virus
iPSC: induced pluripotent stem cell
IFN-I: type I interferon
IFN-III: type III interferon
IFN-λ1: interferon lambda 1
IL: Interleukin
MERS-CoV: Middle East respiratory syndrome coronavirus
MPA: mycophenolic acid
NF-κB: nuclear factor kappa beta
NPC: neural progenitor cell
PSC: pluripotent stem cell
QNHC: quinacrine dihydrochloride
SARS-CoV-2: severe acute respiratory syndrome coronavirus 2
RSV: respiratory syncytial virus
TMPRSS2: transmembrane serine protease 2
TNF: tumor necrosis factor
ZIKV: Zika virus
